# Patterns of X-Linked Retinitis Pigmentosa Genetic Testing in England and Implications for Service Provision

**DOI:** 10.1016/j.xops.2026.101180

**Published:** 2026-04-01

**Authors:** Sol Yates, William Whittaker, Mark Harrison, Stuart Bayliss, Stephanie Barton, Panagiotis I. Sergouniotis, Katherine Payne, Graeme Black

**Affiliations:** 1 Manchester Centre for Health Economics, Division of Population Health, Health Services Research & Primary Care, School of Health Sciences, Faculty of Biology, Medicine and Health, The University of Manchester, Manchester, UK; 2 Public Health, Policy & Systems, University of Liverpool, Liverpool, UK; 3 Faculty of Pharmaceutical Sciences, University of British Columbia, Vancouver, British Columbia, Canada; 4 Centre for Advancing Health Outcomes, St. Paul’s Hospital, Vancouver, British Columbia, Canada; 5 North West Genomic Medicine Service Alliance, Saint Mary’s Hospital, Manchester University NHS Foundation Trust, Manchester M13 9WL, UK; 6 Manchester Centre for Genomic Medicine, Saint Mary’s Hospital, Manchester University NHS Foundation Trust, Manchester M13 9WL, UK; 7 Division of Evolution, Infection and Genomics, School of Biological Sciences, Faculty of Biology, Medicine and Health, University of Manchester, Manchester M13 9P, UK

**Keywords:** Genetic Testing, Inherited retinal diseases, Retinitis pigmentosa, X-linked retinitis pigmentosa, Population prevalence

## Abstract

**Purpose:**

To establish the prevalence of retinitis pigmentosa GTPase regulator (*RPGR*)-associated X-linked retinitis pigmentosa (XLRP) in England.

**Design:**

A cross-sectional prevalence study.

**Participants:**

English National Health Service patients referred for XLRP molecular testing between 2004 and 2024.

**Methods:**

We calculated the overall prevalence rate of *RPGR*-XLRP for the study period by summing the genetically confirmed cases held in a database by a single testing center, which was the sole provider of Open Reading Frame 15 (ORF15) *RPGR* testing in the United Kingdom, in the study period (2004 to 2024). We used this information to calculate a mortality-adjusted minimum prevalence of *RPGR*-XLRP per 100 000 population (2024) in England. We also investigated trends in genetic testing over time and explored equity of access by region and socio-economic status through comparisons of absolute test numbers and testing rates per 100 000 population.

**Main Outcome Measures:**

Prevalence of *RPGR*-XLRP per 100 000 population, overall and stratified by region and Index of Multiple Deprivation (IMD) quintile.

**Results:**

The estimated mortality-adjusted prevalence of *RPGR*-XLRP in England is 1.67 per 100 000 population (2024), corresponding to an estimated 977 living patients in 2024 from 1024 diagnoses made between 2004 and 2024. The estimated mortality-adjusted prevalence of *RPGR*-XLRP among males is 2.18 per 100 000 males (n = 626) and 1.17 per 100 000 females (n = 351) (2024). The mean age of test-positive patients was 44.1 years at study end date (2024). Population-standardized testing rates were broadly consistent across regions, with all regions falling within 19% of the national average, except the North West (34%), reflecting interregional referral patterns. Testing volumes (absolute numbers) per socio-economic quintile (IMD) were within 17% of the average. The number of individuals tested for ORF15 seemed to reduce after the introduction of whole genome sequencing (2019).

**Conclusions:**

We identified an estimated 977 living patients with *RPGR*-associated XLRP from 2594 tested individuals, representing a large absolute number and significant population who could benefit from emerging gene therapy, with prevalence higher than other treatable inherited retinal dystrophies. We found no systematic inequities in test access by socio-economic status or region, though a decline in testing since 2020 warrants further investigation.

**Financial Disclosures:**

Proprietary or commercial disclosure may be found in the Footnotes and Disclosures at the end of this article.

Gene therapy has emerged as a promising treatment for inherited retinal dystrophies (IRDs), with voretigene neparvovec for retinal pigment epithelium-specific 65 kDa protein (*RPE65*)-associated IRD establishing a successful precedent for subsequent monogenic treatments aimed at preserving vision loss by slowing down retinal degeneration over the longer term.^1^ Equitable access to health care on the basis of need is a core aim for many health systems, including the National Health Service (NHS) in England, requiring accurate identification of the need for services.^2^ Newer gene therapies targeting larger patient populations (such as those for retinitis pigmentosa GTPase regulator-associated X-linked retinitis pigmentosa [*RPGR*-XLRP]) will require both an effective estimate of potential treatable population and scalable, equitable access to genetic diagnosis, as confirmation of *RPGR* mutations is a prerequisite for treatment eligibility. Without such equitable diagnostic access, these gene therapies risk being distributed inequitably despite their potential to address broader patient populations.

Inherited retinal dystrophies, caused by pathogenic variants in >260 genes, ultimately result in vision loss through photoreceptor degeneration.^3^^,^^4^ Retinitis pigmentosa (RP), the most common subtype of inherited retinal disease,^5^ is a genetically and clinically diverse condition marked by the onset of night blindness, followed by peripheral vision loss and, ultimately, progressive loss of central vision.^6^ Retinitis pigmentosa affects an estimated 20 to 33 individuals per 100 000 worldwide,^7^^,^^8^ with X-linked RP (XLRP), one of the most common and severe subtypes of RP,^5^ estimated to account for approximately 10% to 20% of cases.^9^, ^10^, ^11^ Pathogenic variants in the *RPGR* gene account for >70% of XLRP cases,^5^^,^^10^^,^^12^ with most occurring in a mutational hotspot known as Open Reading Frame 15 (ORF15), a retina-specific terminal exon of the *RPGR* gene.^12^ The highly repetitive sequence of *RPGR*’s ORF15 region poses a significant challenge for current genetic testing strategies for IRDs,^12^ which is especially relevant given that mutations in the ORF15 region are responsible for approximately half of XLRP cases.^13^ While whole genome sequencing (WGS) is increasingly employed as first-line testing for suspected IRDs,^14^ its technical limitations in sequencing the highly repetitive ORF15 region create a diagnostic gap of incomplete *RPGR* screening and misclassified diagnoses.^12^

This diagnostic gap exemplifies broader systemic challenges in genetic testing delivery. Fragmented referrals, variation in provision, and limited capacity undermine equitable access to comprehensive genetic testing,^14^^,^^15^ potentially preventing not only identification of patients who would be eligible for gene therapies but also limiting access to other important benefits of genetic diagnosis including family planning counseling, cascade screening of relatives, prognosis information, and enrollment in clinical trials.

To predict the impact on the NHS if a gene therapy is approved, it is important to understand prevalence; provided diagnostic pathways have reliably identified eligible patients. For gene therapies to reach their potential, Gavan et al^16^ argue that health systems should jointly evaluate genomic diagnostic tests and gene therapies as a single intervention to accurately assess their full economic impact and opportunity cost, recognizing that accurate diagnosis is essential for the effectiveness of gene therapy. However, the high upfront costs and significant uncertainty regarding long-term effects of gene therapies challenge conventional health technology assessment approaches.^17^

There is limited understanding of the prevalence of *RPGR*-XLRP and trends in genetic testing delivery over time, particularly regarding equitable access. This challenge pervades international health care systems, with significant heterogeneity between national strategies for ophthalmic genetic testing.^14^ One study,^18^ conducted prior to several structural changes in genetic testing—including the formalization of genetic testing in England in 2018^15^—found regional variation in genetic testing rates for a subset of IRDs. The current study analyzes 2 decades of *RPGR* ORF15 testing data (2004 to 2024) to estimate a minimum prevalence of *RPGR*-XLRP diagnosis, describe trends in testing over time, and explore equity of access.

## Methods

### Data Sources

This retrospective analysis examines 2 decades (2004 to 2024) of genetic test data from the Manchester Centre for Genomic Medicine (MCGM), England’s sole provider of the *RPGR* ORF15 genetic test for confirmed XLRP diagnoses since 2003. The diagnostic database represents an ideal source for data as it contains what is likely the single largest grouping of patients with *RPGR*-XLRP in the NHS. During the study period, the test remained technically unchanged despite evolving funding and delivery mechanisms. Initially charity-funded, the test moved to local clinician-led funding from 2010, and subsequently to central NHS Genomic Medicine Service funding from 2018.^15^^,^^18^ As of 2020, the test corresponds to the R32 panel test for possible XLRP in the national genomic test directory.^19^ All patients under the care of the NHS who underwent *RPGR* ORF15 testing at MCGM between January 2004 and September 2024 were included. Testing requests comprised 2 categories: (1) diagnostic testing in the proband for the first affected family member seeking medical attention for a genetic disorder with an unknown mutation, and (2) cascade tests in relatives where the familial mutation is already known, including carrier, predictive or diagnostic tests. This project was conducted as a clinical audit and service evaluation using deidentified genetic testing data. The University of Manchester Ethics Decision Tool determined that formal ethical approval was not required for this retrospective analysis. This study adhered to the tenets of the Declaration of Helsinki. No studies involving human or animal participants were conducted by the authors for this article. This study adheres to the Strengthening the Reporting of Observational Studies in Epidemiology (STROBE) reporting guidelines (Supplemental Table S1, available at www.ophthalmologyscience.org).

### Inclusion and Exclusion Criteria

Although a small number of tests were conducted prior to 2004 (n = 35, of which 17 were positive), these are excluded from our sample frame due to the limited availability and inconsistency of testing during that period. We acknowledge that this may result in a slight underestimation of the true number of *RPGR*-positive cases. Exclusion criteria comprised individuals with invalid dates of birth (n = 26); nonstandard sex codes (e.g., fetal testing, often coded as “other”) (n = 32), non-England residents (n = 1438), tests outside the study period, and repeat tests for the same individual. We also excluded all tests conducted solely for research purposes, without direct clinical implications, to ensure the clinical relevance of our findings (i.e., all included tests were performed with the intent to inform health care decisions). A genetic ophthalmologist (G.B.) reviewed all test records to classify cases as probands or carriers and to confirm *RPGR*-XLRP positive results.

### Data Extraction

Initial laboratory data contained date test received; laboratory number; residential postcode; sex; and test description and genotype. Sex was determined based on biological sex recorded in medical records at the time of diagnosis, which may not reflect participants’ gender identity. To ensure anonymity, patient postcodes were converted to 2011 Census Lower Super Output Areas, which are statistical neighborhoods of approximately 1500 residents. After linkage, all individually identifying fields were removed. We excluded patients residing outside of England, as the postcode mapping tool^20^ is limited to English addresses. We linked Lower Super Output Area data with 2019 Index of Multiple Deprivation (IMD) quintiles;^21^ Government Office Regions using Office for National Statistics lookup files^22^; and Office for National Statistics mid-year population estimates from 2004 to 2024^23^ for population rate calculations (the latest year available). Minor adjustments were made to <10 of 32 000 Lower Super Output Areas to align 2021 census population data (for years 2021-2024; the latest years with population data) with 2019 IMD data. Data extraction and coding were performed using R 4.4.1 (R Foundation for Statistical Computing).^24^

### Analysis

The dataset contains data from patients under the care of the NHS who underwent testing between 2004 and 2024 at MCGM. Analyses were conducted at the patient level.

### Prevalence of RPGR-XLRP

To provide a point prevalence for the year 2024, we adjusted our case numbers for mortality. We estimated the number of living patients as of September 2024 by applying age-specific and sex-specific mortality rates from the UK National Life Tables (2022-2024)^25^ to our cohort. This estimated number of living patients was then divided by the 2024 mid-year population estimate for England to calculate the mortality-adjusted prevalence. We stratified patients into age groups relevant to NHS service planning: 0 to 18 years; 19 to 39 years; 40 to 64 years; and ≥65 years. We also estimated the overall test positivity rate as the percentage of positive results among all individuals tested, and the proband test positivity rate as the percentage of probands with a positive result. Our data likely represents an underestimate of the true prevalence of the disease in England; we only included individuals who presented to MCGM, excluding those referred to other specialist ophthalmic genetic testing centers, such as Oxford (from 2015) and London (from 2018). We also excluded positives tested prior to 2004 when testing was sporadic.

### Trends in Testing over Time

To describe trends in testing over time, we examined trends in testing volumes using descriptive statistics, including annual testing volumes, positive diagnosis rates, and population-adjusted testing rates per 100 000 individuals. To highlight changes over time, we applied 5-year rolling averages and compared average testing volumes before and after potential change points. To examine the potential impact of the coronavirus disease 2019 pandemic on testing patterns, we compared average annual testing volumes before 2020 (2004 to 2019) with those from 2020 onward (2020 to 2024). We also examined the number of individuals tested by age at study end (2024) to understand the age distribution of testing over time (Supplemental Figure S1, available at www.ophthalmologyscience.org).

### Equity of Access

To explore equity of access to *RPGR*-XLRP testing, we conducted 3 analyses. First, we compared testing rates per 100 000 population (2004 to 2024) across government office regions to identify geographic disparities while accounting for different regional population sizes. Second, we compared absolute testing numbers across IMD quintiles to assess socio-economic disparities, using absolute numbers since IMD quintiles contain approximately equal population sizes by design. Third, we calculated testing rates per 100 000 population (2004 to 2024) for IMD quintiles within each region to examine the intersection of geographic and socio-economic factors. We interpreted similar testing rates across regions and socio-economic groups as evidence of equitable access, consistent with NHS policy recommendations to provide “consistent and equitable care” across England’s population through the Genomic Medicine Service.^2^ However, this interpretation assumes disease prevalence is uniform across groups, an assumption that is likely unrealistic. Substantial variations in testing rates could indicate potential barriers to access, though we acknowledged that some variation might reflect differences in referral patterns or local clinical practices rather than inequitable access. A sensitivity analysis investigated equity of access by socioeconomic status and geographical region further using indirect standardization (Supplemental Tables S2-S3, available at www.ophthalmologyscience.org).

## Results

### Prevalence of RPGR-XLRP

The final dataset includes data on 2594 individuals who underwent testing between 2004 and 2024. After adjusting for mortality, we estimated that 2458 patients (94.8%) were alive in 2024, of whom 977 had tested positive (95.5% of the 1024 positive cases). This yields a mortality-adjusted point prevalence of 1.67 cases per 100 000 population (2024) (Table 1). Among the 657 positive males identified, the estimated number of living patients is 626 for a mortality-adjusted prevalence of 2.18 per 100 000 males, while for the 367 positive females, the estimated number of living patients is 351 for a prevalence of 1.17 per 100 000 females. A sensitivity analysis using standardized mortality ratio-adjusted life tables estimates 949 positive patients alive at study end (see Supplemental Table S4 for calculation, available at www.ophthalmologyscience.org).

**Table 1:** Demographics and Prevalence of RPGR-XLRP (2004-2024)

Characteristic	Value
Patient demographics	
Total patients tested, n	2594
Total positive cases, n	1024
Age distribution	
Current age (2024), mean ± SD (range)	45.3 ± 19.7 (3.0 - 110.0)
Age at testing, mean ± SD (range)	34.8 ± 18.9 (0.0 - 91.0)
Current age of positives (2024), mean ± SD (range)	44.1 ± 19.3 (4.0 - 110.0)
Age at testing of positives, mean ± SD (range)	34.0 ± 17.9 (0.0 - 89.0)
Time since positive diagnosis (years), mean ± SD	10.1 ± 6.1
Sex distribution of testing	
Male, n (% of total)	1786 (68.9%)
Female, n (% of total)	808 (31.1%)
Sex distribution of RPGR-XLRP positive	
Male positives, n (% of total positives)	657 (64.2%)
Female positives, n (% of total positives)	367 (35.8%)
Estimated prevalence per 100 000 population (2024) (95% CI)	
Overall	1.67 (1.56 - 1.77)
Male	2.18 (2.01 - 2.35)
Female	1.17 (1.05 - 1.30)

CI = confidence interval; RPGR-XLRP = retinitis pigmentosa GTPase regulator gene–associated X-linked retinitis pigmentosa; SD = standard deviation.

Percentages are based on the total number of patients tested unless otherwise indicated. Prevalence estimates are per 100 000 population (95% CI), calculated for the population in 2024. Current age calculated as of September 17, 2024.

The mean age at testing positive was 34 years, with patients ranging from 0 to 91 years old at the time of diagnosis. As of study end (2024), test-positive patients in our dataset have a mean current age of 44.1 years, ranging from 4 to 110 years old. Table 2 shows that most positive cases are working-age individuals, with 427 (42%) in the 40 to 64 years age group and 335 (33%) in the 19 to 39 years age group. Together, 74% of patients are currently of working age (19 to 64 years). The proportion currently in the 0 to 18 years age group is low (n = 104 cases, 10%), but there was a recent trend toward more diagnoses in younger populations (Supplemental Figure S1).

**Table 2:** Age and Sex Distribution of RPGR-XLRP Positive Cases (2004-2024)

Age Group	Male, n (%)	Female, n (%)	Total, n (%)
0-18 years	89 (8.7%)	15 (1.5%)	104 (10.2%)
19-39 years	225 (22.0%)	110 (10.7%)	335 (32.7%)
40-64 years	243 (23.7%)	184 (18.0%)	427 (41.7%)
65+ years	100 (9.8%)	58 (5.7%)	158 (15.4%)
Total	657 (64.2%)	367 (35.8%)	1024 (100.0%)

n = number; RPGR-XLRP = retinitis pigmentosa GTPase regulator X-linked retinitis pigmentosa.

Percentages represent proportion of total cohort. Age calculated as of September 17, 2024. Totals may not sum to 100% due to rounding.

### Trends in Testing over Time

**Figure 1:**
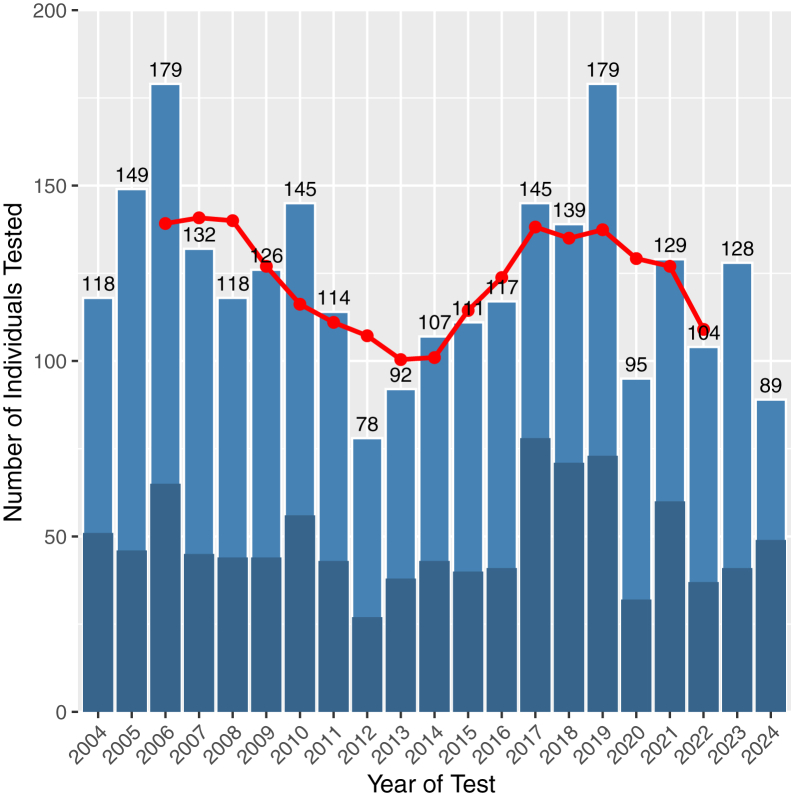
Number of individuals tested by year in England, 2004-2024. The bars show the total number of individuals tested each year, with the exact count displayed above. The darker blue portion of each bar represents the number who tested positive. The red line indicates the 5-year moving average of total tests (2006-2022).

Testing volumes were relatively stable until 2019 but showed evidence of a decline thereafter (Fig 1). Prior to 2020, an average of 128 individuals were tested per year, compared with 109 per year from 2020 onward. We calculated an overall test positivity rate of 39% (1024 positives out of 2594 tested). The overall national testing rate was 4.43 per 100 000 population (2024) (Table 3). Among probands, the positivity rate was 29% (519 positive probands out of 1811 tested). Test positivity rates remained broadly similar over time, though proband positivity decreased after 2019, coinciding with the drop in testing volumes (Supplemental Figure S2-S3, available at www.ophthalmologyscience.org). Sex-specific analysis revealed differences in positivity rates. Males showed an overall positivity rate of 37%, with a proband positivity rate of 28% (410 of 1440 tested). Females had a higher overall positivity rate of 45% and a similar proband positivity rate of 29% (109 of 371 tested).

**Table 3:** Overall Test Positivity (2004-2024)

Sex	Total Tested	Total Proband	Total Positives (Positivity Rate)	Proband Positives (Positivity Rate)	Testing Rate per 100 000 (2004-2024)
Overall	2594	1811	1024 (39%)	519 (28.66%)	4.78
Male	1786	1440	657 (37%)	410 (28.47%)	6.69
Female	808	371	367 (45%)	109 (29.38%)	2.93

Positivity rate is calculated as the proportion of positive cases among those tested within that category. Testing rate = number of individuals tested per 100 000 population.

### Equity of Access

**Figure 2:**
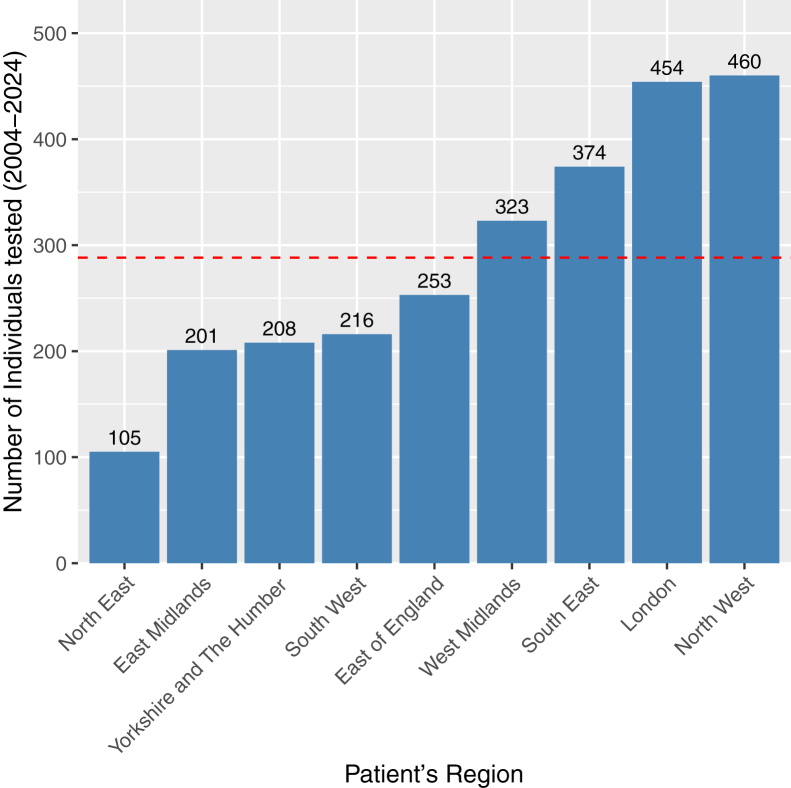
Total number of individuals tested by region in England, 2004-2024. The bars represent the total number of individuals tested in each region over the entire period, with the exact count displayed above. The red dashed line indicates the national average.

**Figure 3:**
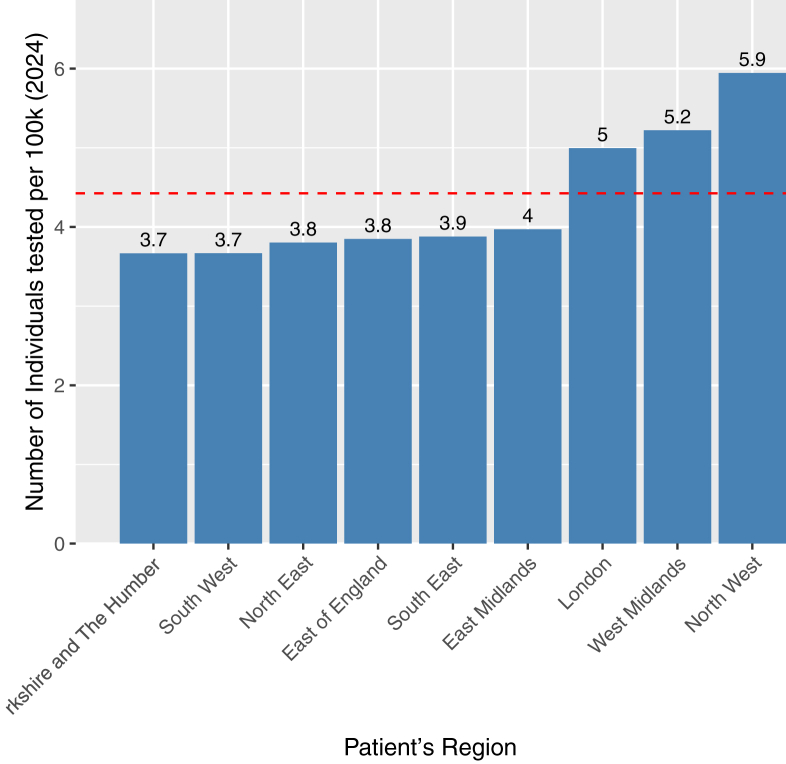
Average testing rate by region in England, 2024. The bars represent the number of individuals tested per 100 000 population (2024) for each region over the entire period, with the exact rate displayed above. The red dashed line represents the national average rate. Rates are based on testing data from 2004-2024 and the population from 2024.

**Figure 4:**
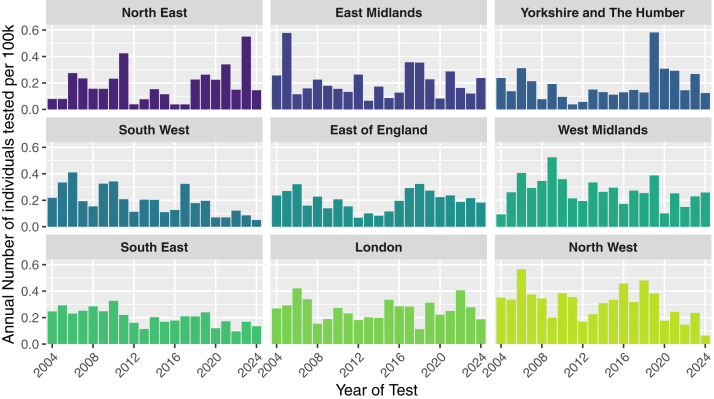
Annual testing rates by region in England, 2004-2024. The bars represent the annual rate of individuals tested per 100 000 population (2004-2024) for each region. Rates are based on testing data from 2004-2024 and the average population from 2004-2024.

**Figure 5:**
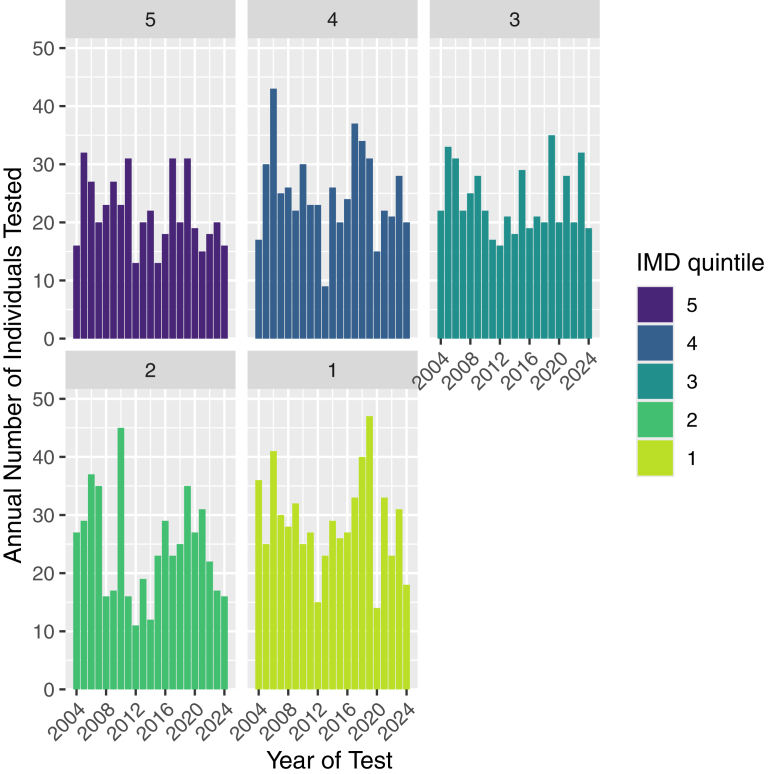
Annual total number of individuals tested by IMD quintile in England, 2004-2024. The bars show the total number of individuals tested in each IMD quintile per year. Based on testing data from 2004-2024. IMD = Index of Multiple Deprivation.

**Figure 6:**
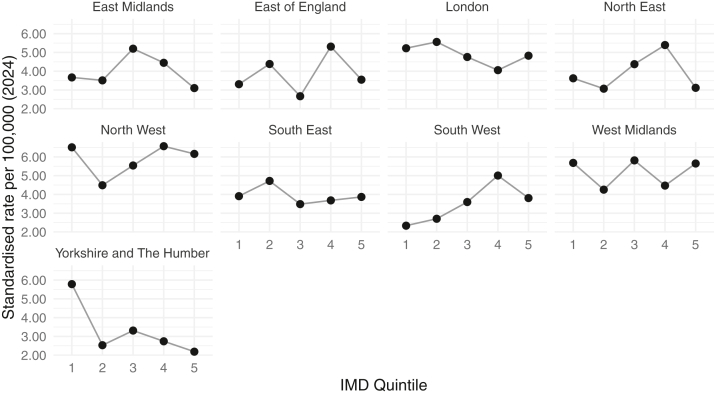
Population-standardized testing rates by IMD quintile and region in England (2024). Each panel shows average annual testing rates per 100 000 population across IMD quintiles within a region. Points indicate IMD quintile-specific rates and lines connect quintiles to illustrate within-region trends. Rates are calculated using testing data from 2004-2024 and the population from 2024. IMD = Index of Multiple Deprivation.

Testing volumes varied across regions, ranging from 105 in the North East to 460 in the North West (Fig 2). However, when standardized by population size, testing rates were generally uniform across regions, with all regions except the North West (5.95 tests per 100 000 [2024]) falling within 19% of the national average of 4.43 per 100 000 population (2024) (Fig 3). The North West contributed most to testing numbers prior to 2017 (Fig 4). At the national level, testing volumes were relatively evenly distributed across all 5 IMD quintiles, which are population-balanced by design, with all quintiles falling within 17% of the national average (Fig 5). Within individual regions, where IMD quintiles are not population-balanced, testing rates showed more variation across socioeconomic groups, though without a systematic pattern indicative of clear inequality (Fig 6). A sensitivity analysis using indirect standardization showed similar findings, with IMD quintiles within 11% of the expected rates, and all regions within 20% of the rate, except the North West (34%) (Supplemental Tables S2-S3).

## Discussion

This study investigated the uptake of, and trends in access to, *RPGR* ORF15 genetic testing within the NHS in England between 2004 and 2024. Using data from a single national testing center, we estimate a mortality-adjusted minimum prevalence of 1.67 per 100 000 population (2024), reflecting the estimated living genetically confirmed cohort from a single center. As this center represents 1 of 3 currently providing genetic testing for IRDs in England, this estimate likely represents a lower bound of the true national prevalence. Our estimate should be considered a minimum prevalence for 3 possible reasons. It is derived from a single testing center and captures only genetically confirmed cases from that site. X-linked RP may be misclassified as other types of RP in clinical practice, potentially leading to underascertainment. First-line WGS for suspected XLRP will not detect mutations within the ORF15 mutation hotspot, resulting in missed diagnoses. These factors suggest our prevalence estimate is conservative, though the true prevalence remains unknown. With 74% of cases in working age individuals (19 to 64), this population could have considerable implications for health economic evaluations of future gene therapies.

We observed relatively consistent *RPGR* ORF15 testing volumes throughout the study period until 2019, with an average of 128 tests per year. Importantly, testing rates remained broadly similar across geographic regions and socioeconomic strata, suggesting that access to genetic testing has not been systematically affected by socio-economic status or region at the national level. This pattern of similar testing rates is notable given the significant funding evolution during our study period, as *RPGR* testing transitioned from charity-based funding to local commissioning arrangements, and finally to centralized national commissioning from 2018. Despite these organizational upheavals, we found generally uniform testing rates within regions, deprivation quintiles, and deprivation quintiles within regions. However, previous research from the UK found that children from socio-economically disadvantaged backgrounds and ethnic minority groups experience higher rates of low and no vision certification.^26^ Because ethnicity data were not recorded in our database, we cannot verify whether similar disparities exist in the *RPGR*-XLRP population. This limitation highlights the need for further investigation into whether structural or service-level factors may influence referral patterns.

After 2019, we observed a notable 15% decline in testing volumes (from 128 to 109 tests per year) accompanied by decreasing proband positivity rates. This decline appears to result from 3 converging factors. First, the coronavirus disease 2019 pandemic disrupted health care services and referral patterns, highlighting the vulnerability of specialized genetic testing services to system-wide pressures. Second, the implementation of WGS programs in Oxford (2015) and London (2018) may have diverted referrals to alternative testing pathways not captured in our Manchester database. Third, the broader shift toward next-generation sequencing (NGS) technologies from 2012 onward,^27^ while improving overall IRD diagnostic capacity, may have reduced *RPGR*-XLRP detection rates where complementary targeted ORF15 testing was not routinely performed. Standard short-read WGS or panel-based NGS as first-line testing risks missing pathogenic variants within the low-complexity ORF15 exon of *RPGR*, which is prone to low coverage and alignment failure due to its highly repetitive, purine-rich sequence. In RP cohorts that were negative on standard NGS, supplementary ORF15-specific testing identified additional pathogenic variants in 14% to 31% of cases,^28^^,^^29^ reflecting the diagnostic value of reflex testing the ORF15 region, rather than the proportion of all ORF15 variants missed by NGS. In our cohort, targeted polymerase chain reaction amplification and Sanger sequencing of ORF15 (see Supplementary Methods of Shu et al^30^) had a diagnostic rate of 39% overall and 29% for probands, demonstrating the continued clinical value of targeted ORF15 testing. Without systematic reflex ORF15 testing for inconclusive genetic test results or coordinated pathways between the 3 IRD testing centers, substantial numbers of patients may remain undiagnosed.

These findings have several important implications for the implementation of gene therapies for IRDs. Our empirically derived, mortality-adjusted minimum prevalence estimate of 1.67 per 100 000 population (2024) indicates that *RPGR*-XLRP affects a substantially larger patient population than *RPE65*-associated IRD, for which a gene therapy has already received regulatory approval.^31^ Given the multiple sources of underascertainment, this estimate is likely a lower bound of the true national burden. Consistent with prior literature,^11^ the true prevalence of *RPGR*-XLRP may exceed the ultrarare disease threshold of 2 per 100 000.^32^^,^^33^ If true prevalence does exceed this threshold, *RPGR*-XLRP could represent one of the larger genetically defined patient groups within the IRD spectrum, with our single-center data alone identifying an estimated 977 genetically confirmed cases. While commercial interest in ultrarare diseases is often limited by small patient populations and challenges in recouping development costs,^34^ even at our minimum observed prevalence of 1.67 per 100 000, the patient population may be sufficient to support therapeutic development. Although patients eligible for gene therapy will be a subset of the total *RPGR*-XLRP population, the prevalence observed in this study already exceeds the population sizes implicitly assumed in previous health technology assessments for *RPE65*-associated IRD gene therapy, where the National Institute for Health and Care Excellence estimated that there were between 57 and 564 individuals affected individuals in England, of whom approximately 86 were considered eligible for treatment.^35^ Expressed as a population rate, this corresponds to an implied prevalence of approximately 0.1 to 1.0 per 100 000 population (2019). Our observed minimum prevalence of 1.67 per 100 000 (2024) for genetically confirmed *RPGR*-XLRP cases exceeds this range.

It is important to note that not all the estimated 977 positive cases represent immediate gene-therapy candidates. The therapeutic landscape for *RPGR*-associated retinopathy is evolving with Beacon Therapeutics' laruparetigene zovaparvovec (laru-zova) having completed enrollment in its phase II/III VISTA trial with topline results expected in late 2026, reflecting ongoing efforts to bring targeted treatments to patients with *RPGR*-XLRP.^36^ In contrast, MeiraGTx/Janssen’s botaretigene sparoparvovec did not meet its primary endpoint in the phase III LUMEOS trial in 2025, underscoring the challenges of demonstrating clinically meaningful benefit in late stage studies.^37^ Beyond gene-specific therapies, gene-agnostic therapies under investigation may broaden future treatment options regardless of the underlying mutation.^38^

Our observations of genetic testing trends indicate that targeted ORF15 testing as a reflex test would complement current sequencing approaches, which, despite substantial advances, still identify causative variants in only 50% to 75% of patients with IRD.^39^ The predominance of working-age individuals (74% aged 19 to 64 years) in our cohort represents potential labor market and societal benefits from effective treatments that conventional cost-effectiveness analyses may not fully capture.^16^^,^^17^ Similar challenges in genetic testing infrastructure and service fragmentation exist across health care systems globally,^14^^,^^15^ suggesting that widespread underdiagnosis may be replicated internationally. This pattern potentially prevents not only identification of patients eligible for gene therapies but also limits access to other important benefits of genetic diagnosis, including family planning counseling, cascade screening of relatives, prognosis information, and enrollment in clinical trials.^14^

This study has several important limitations. Our analysis draws on data from a single national testing center. Although this center was the sole provider of *RPGR* testing for many years, the subsequent establishment of WGS programs in Oxford and London means our data does not capture all tests performed in England. This redistribution of testing likely explains much of the post-2019 decline in volumes at our center and leads to underestimation of true national prevalence. Additionally, we excluded some tests conducted before 2004, further contributing to potential underestimation of both historical and current case numbers.

Our reliance on patient referrals means we likely underestimate true disease prevalence, as many patients may seek testing at other centers or remain undiagnosed. Importantly, no systematic mechanism exists to identify *RPGR*-XLRP cases among inconclusive results from the Oxford and London WGS programs. However, one limitation introduces uncertainty in both directions: while we applied national life tables to estimate mortality, our database lacks individual patient-level mortality data. Our mortality adjustment assumes that patients with *RPGR*-XLRP experience similar age-specific and sex-specific mortality rates to the general population. If *RPGR*-XLRP patients have higher mortality due to associated complications or comorbidities, we may overestimate the current living population; conversely, if their mortality is similar to or lower than the general population, our estimates would be more accurate. Available mortality data from people with RP in Korea suggest standardized mortality ratios may differ from the general population.^40^ A sensitivity analysis applying these standardized mortality ratio-adjusted life tables to our cohort estimates 949 positive patients alive at study end, compared with 977 using general population life tables, suggesting this approach provides estimates similar to the general-population life table approach. On balance, these limitations suggest our findings more likely represent a conservative estimate of true disease burden rather than an inflated one.

Despite these limitations, this study likely represents the most complete and empirical prevalence estimate of *RPGR*-XLRP conducted in England to date. Key strengths include its national scope, 20-year study period, focus on the primary *RPGR* ORF15 testing provider, and the large sample size of 2594 tests providing robust characterization of testing patterns and outcomes. Our findings of 1.67 per 100 000 can be contextualized against previous literature-review-based studies reporting prevalence estimates of 2.7 to 3.5 per 100 000 males in the United States, Europe, and Australia.^11^ However, direct comparison is limited as those studies used clinical inheritance pattern assignment with misclassification adjustments, while ours reports genetically confirmed cases. International testing accessibility and diagnostic capacity limitations may contribute to underascertainment in multiple settings.

This study provides an empirically derived, genetically confirmed prevalence estimate for *RPGR*-XLRP in England. While testing patterns remained consistent over 2 decades, the post-2019 decline may possibly reflect redistribution of referrals to Oxford and London WGS programs rather than reduced diagnostic activity overall. However, the limitations of standard short-read WGS in detecting ORF15 variants suggest some patients may remain undiagnosed without complementary targeted testing. As gene therapy for *RPGR*-XLRP approaches clinical availability, understanding the true prevalence will be important for equitable access. Our minimum prevalence of 1.67 per 100 000 suggests *RPGR*-XLRP affects a patient population larger than *RPE65*-associated IRD, which has an approved gene therapy, though true prevalence may possibly exceed ultrarare thresholds if underascertainment is substantial. A substantial patient population potentially supports the case for therapeutic development. Realizing equitable access to future treatments requires proactive strategies to identify and prepare treatment-eligible patients through systematic integration of ORF15 reflex testing, coordinated pathways between testing centers, and recognition of specialized services’ vulnerability to health care disruptions. While we found no evidence of inequitable access to genetic testing across regions and socio-economic groups, this interpretation assumes disease prevalence is uniform across these groups, which may not necessarily be the case (Supplemental Figure S4, available at www.ophthalmologyscience.org). Although substantial variations in testing rates could indicate potential barriers to access, some variation may reflect differences in referral patterns or local clinical practices rather than inequitable access. Therefore, maintaining equitable access remains important, given the growing importance of genetic testing for accessing gene-specific therapies and clinical trials.
