# Supplementary material for: Patterns of X-Linked Retinitis Pigmentosa Genetic Testing in England and Implications for Service Provision
Source: Ophthalmol Sci. 2026 Apr 1;6(6):101180. doi: 10.1016/j.xops.2026.101180 (PMC13127330; doi:10.1016/j.xops.2026.101180)
Supplement: Supplemental Figure S1 [file mmc1.pdf]

Supplemental Figure S1**.** Annual testing rates by age group in England, 2004-2024.

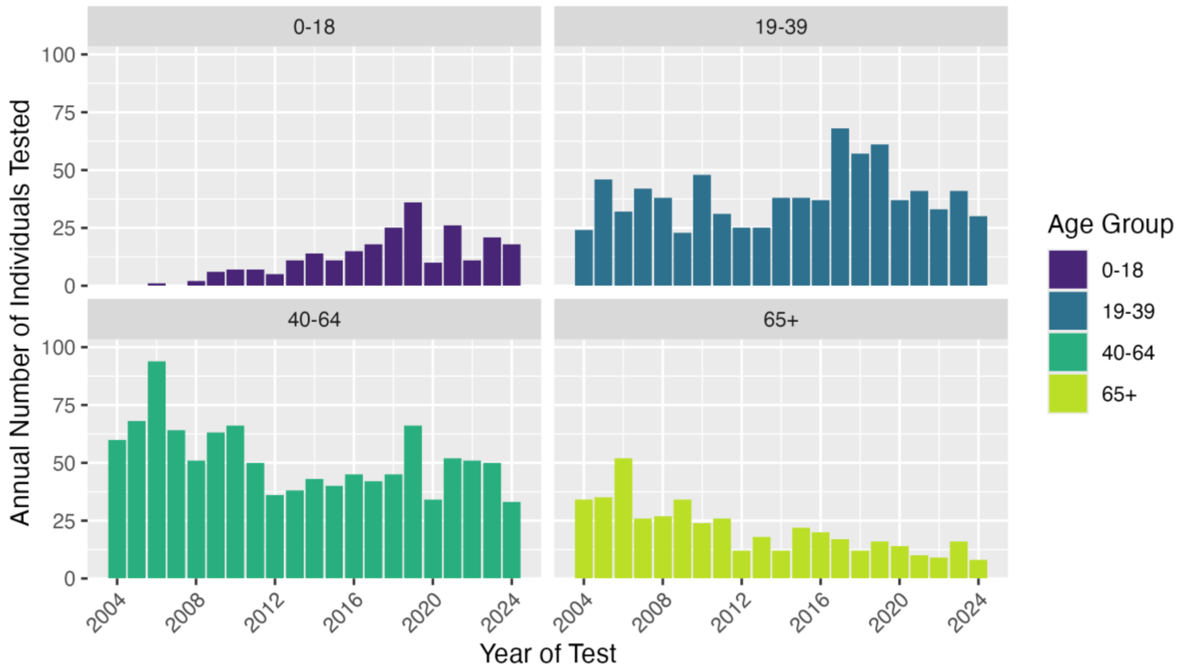

The bars represent the annual number of individuals tested for each age group. Based on testing data from 2004-2024.
