# Supplementary material for: Patterns of X-Linked Retinitis Pigmentosa Genetic Testing in England and Implications for Service Provision
Source: Ophthalmol Sci. 2026 Apr 1;6(6):101180. doi: 10.1016/j.xops.2026.101180 (PMC13127330; doi:10.1016/j.xops.2026.101180)
Supplement: Supplemental Figure S2 [file mmc2.pdf]

Supplemental Figure S2**.** Annual Test positivity by year, 2004-2024.

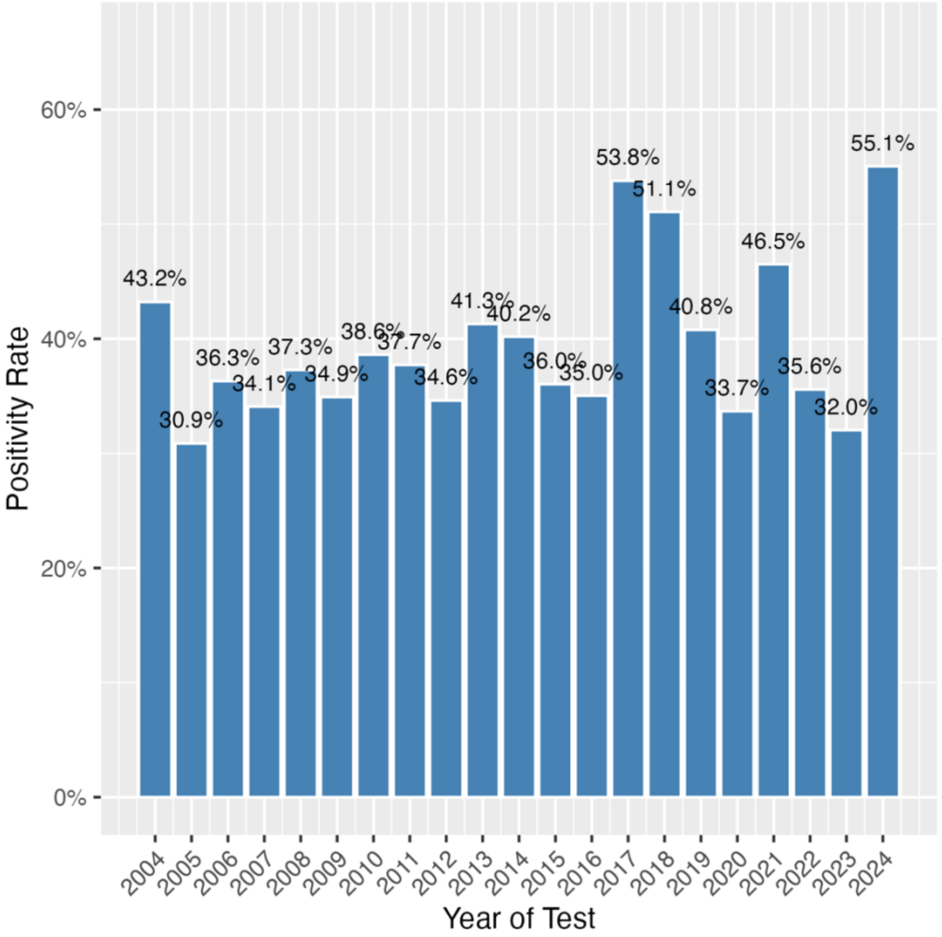

The bars represent the percentage of tests completed in a year, in which the individual tested positive for *RPGR*-XLRP. Based on testing data from 2004-2024.
