# Supplementary material for: Patterns of X-Linked Retinitis Pigmentosa Genetic Testing in England and Implications for Service Provision
Source: Ophthalmol Sci. 2026 Apr 1;6(6):101180. doi: 10.1016/j.xops.2026.101180 (PMC13127330; doi:10.1016/j.xops.2026.101180)
Supplement: Supplemental Figure S4 [file mmc4.pdf]

Supplemental Figure S4**.** Overall Test positivity by Region, 2004-2024.

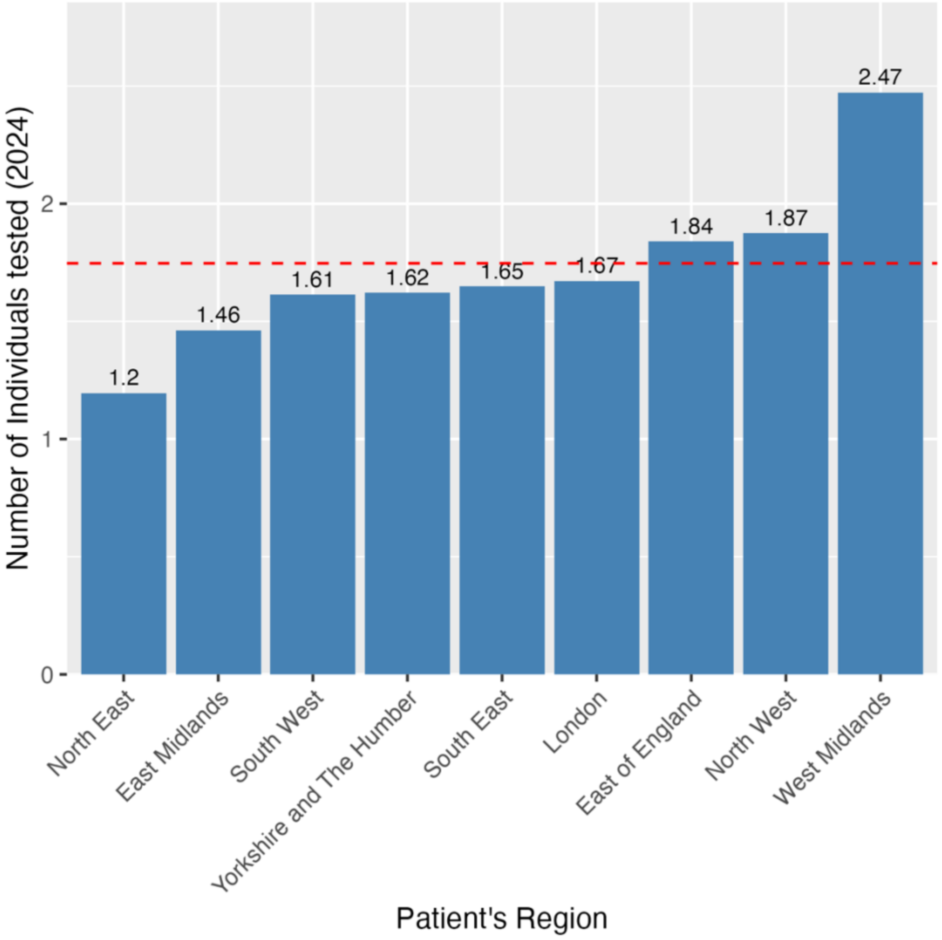

The bars represent the population-standardised rate of individuals testing positive from each Region. Based on testing data from 2004-2024, and population data from 2024. Red line represents national average population-standardised rate of individuals tested.
