# Supplementary material for: Patterns of X-Linked Retinitis Pigmentosa Genetic Testing in England and Implications for Service Provision
Source: Ophthalmol Sci. 2026 Apr 1;6(6):101180. doi: 10.1016/j.xops.2026.101180 (PMC13127330; doi:10.1016/j.xops.2026.101180)
Supplement: Supplemental Table S1 [file mmc5.pdf]

Supplemental Table S1. STROBE Statement—checklist of items that should be included in reports of observational studies

	Item No.	Recommendation	Page No.	Relevant text from manuscript
**Title and abstract**	1	(*a*) Indicate the study's design with a commonly used term in the title or the abstract	3	Cross-sectional prevalence study
		(*b*) Provide in the abstract an informative and balanced summary of what was done and what was found	3	calculate a mortality-adjusted minimum prevalence of *RPGR*-XLRP per 100,000 population (2024) in England
**Introduction**				
Background/rationale	2	Explain the scientific background and rationale for the investigation being reported	4	Gene therapy has emerged as a promising treatment for inherited retinal dystrophies (IRDs) … Without such equitable diagnostic access, these gene therapies risk being distributed inequitably
Objectives	3	State specific objectives, including any prespecified hypotheses	7	The current study analyses two decades of *RPGR* ORF15 testing data (2004 to 2024) to estimate a minimum prevalence of *RPGR*-XLRP diagnosis, describe trends in testing over time, and explore equity of access
**Methods**				
Study design	4	Present key elements of study design early in the paper		

Setting	5	Describe the setting, locations, and relevant dates, including periods of recruitment, exposure, follow-up, and data collection	
Participants	6	(*a*) *Cohort study*—Give the eligibility criteria, and the sources and methods of selection of participants. Describe methods of follow-up<br>*Case-control study*—Give the eligibility criteria, and the sources and methods of case ascertainment and control selection. Give the rationale for the choice of cases and controls<br>*Cross-sectional study*—Give the eligibility criteria, and the sources and methods of selection of participants	
		(*b*) *Cohort study*—For matched studies, give matching criteria and number of exposed and unexposed<br>*Case-control study*—For matched studies, give matching criteria and the number of controls per case	
Variables	7	Clearly define all outcomes, exposures, predictors, potential confounders, and effect modifiers. Give diagnostic criteria, if applicable	
Data sources/ measurement	8*	For each variable of interest, give sources of data and details of methods of assessment (measurement). Describe comparability of assessment methods if there is more than one group	
Bias	9	Describe any efforts to address potential sources of bias	
Study size	10	Explain how the study size was arrived at	

Continued on next page

Quantitative variables	11	Explain how quantitative variables were handled in the analyses. If applicable, describe which groupings were chosen and why		
Statistical methods	12	(*a*) Describe all statistical methods, including those used to control for confounding		
		(*b*) Describe any methods used to examine subgroups and interactions		
		(*c*) Explain how missing data were addressed		
		(*d*) *Cohort study*—If applicable, explain how loss to follow-up was addressed<br>*Case-control study*—If applicable, explain how matching of cases and controls was addressed<br>*Cross-sectional study*—If applicable, describe analytical methods taking account of sampling strategy		
		(*e*) Describe any sensitivity analyses		
**Results**				
Participants	13*	(a) Report numbers of individuals at each stage of study—eg numbers potentially eligible, examined for eligibility, confirmed eligible, included in the study, completing follow-up, and analysed	10	The final dataset includes data on 2594 individuals who underwent testing between 2004 and 2024
		(b) Give reasons for non-participation at each stage	7-8	Exclusion criteria comprised: individuals with invalid dates of birth (n=26); non-standard sex codes (e.g., fetal testing, often coded as "other") (n=32), non-England residents (n=1438), tests outside the study period and repeat tests for the same individual. We also excluded all tests conducted solely for research purposes, without direct clinical implications
		(c) Consider use of a flow diagram		N/A
Descriptive data	14*	(a) Give characteristics of study participants (eg demographic, clinical, social) and information on exposures and potential confounders	10	The mean age at testing positive was 34 years …<br>As of study end (2024), test-positive patients…mean current age of 44.1 years…

		(b) Indicate number of participants with missing data for each variable of interest		Exclusion criteria comprised: individuals with invalid dates of birth (n=26); non-standard sex codes (e.g., fetal testing, often coded as "other") (n=32), non-England residents (n=1438), tests outside the study period and repeat tests for the same individual. We also excluded all tests conducted solely for research purposes, without direct clinical implications
		(c) *Cohort study*—Summarise follow-up time (eg, average and total amount)		
Outcome data	15*	*Cohort study*—Report numbers of outcome events or summary measures over time		
		Case-control study—Report numbers in each exposure category, or summary measures of exposure		
		Cross-sectional study—Report numbers of outcome events or summary measures	*15*	The final dataset includes data on 2594 individuals who underwent testing between 2004 and 2024 This yields a mortality-adjusted point prevalence of 1.67 cases per 100,000 population (2024)
Main results	16	(*a*) Give unadjusted estimates and, if applicable, confounder-adjusted estimates and their precision (eg, 95% confidence interval). Make clear which confounders were adjusted for and why they were included	10	Among the 657 positive males identified …<br>a sensitivity analysis using SMR-adjusted lifetables estimates
		(*b*) Report category boundaries when continuous variables were categorized	9	We stratified patients into age groups relevant to NHS service planning: 0 to 18-years; 19 to 39-years; 40 to 64-years; and 65-years and above
		(*c*) If relevant, consider translating estimates of relative risk into absolute risk for a meaningful time period		N/A

Continued on next page

Other analyses	17	Report other analyses done—eg analyses of subgroups and interactions, and sensitivity analyses	10	A sensitivity analysis using SMR-adjusted lifetables
**Discussion**				
Key results	18	Summarise key results with reference to study objectives	12	Using data from a single national testing centre, we estimate a mortality-adjusted minimum prevalence of 1.67 per 100,000 population
Limitations	19	Discuss limitations of the study, taking into account sources of potential bias or imprecision. Discuss both direction and magnitude of any potential bias	15	This study has several important limitations.
Interpretation	20	Give a cautious overall interpretation of results considering objectives, limitations, multiplicity of analyses, results from similar studies, and other relevant evidence	16	Our reliance on patient referrals means we likely underestimate true disease prevalence,
Generalisability	21	Discuss the generalisability (external validity) of the study results	14	Similar challenges in genetic testing infrastructure and service fragmentation exist across healthcare systems globally
**Other information**				
Funding	22	Give the source of funding and the role of the funders for the present study and, if applicable, for the original study on which the present article is based	1	This work was supported by an unrestricted grant from Janssen Pharmaceuticals, Beerse, Belgium. We also acknowledge the following sources of funding: the Wellcome Trust (224643/Z/21/Z, Clinical Research Career Development Fellowship to P.I.S.); the UK National Institute for Health Research (NIHR) Clinical Lecturer Programme (CL-2017-06-001 to P.I.S.); the NIHR Manchester

		Biomedical Research Centre (NIHR 203308 to P.I.S., G.C.B., KP).

*Give information separately for cases and controls in case-control studies and, if applicable, for exposed and unexposed groups in cohort and cross-sectional studies.

Note: An Explanation and Elaboration article discusses each checklist item and gives methodological background and published examples of transparent reporting. The STROBE checklist is best used in conjunction with this article (freely available on the Web sites of PLoS Medicine at http://www.plosmedicine.org/, Annals of Internal Medicine at http://www.annals.org/, and Epidemiology at http://www.epidem.com/). Information on the STROBE Initiative is available at www.strobe-statement.org.
