# Supplementary material for: Patterns of X-Linked Retinitis Pigmentosa Genetic Testing in England and Implications for Service Provision
Source: Ophthalmol Sci. 2026 Apr 1;6(6):101180. doi: 10.1016/j.xops.2026.101180 (PMC13127330; doi:10.1016/j.xops.2026.101180)
Supplement: Supplemental Table S2 [file mmc6.pdf]

Supplemental Table S2. Age and Sex Standardised Genetic Testing for RPGR-XLRP by Region (2004-2024)

Region	Observed	Expected	STR	CI
North East	105	122.05	0.86	[0.7, 1.04]
North West	460	344.08	1.34	[1.22, 1.46]
Yorkshire and The Humber	208	258.68	0.80	[0.7, 0.92]
East Midlands	201	218.31	0.92	[0.8, 1.06]
West Midlands	323	278.32	1.16	[1.04, 1.29]
East of England	253	276.52	0.91	[0.81, 1.03]
London	454	444.03	1.02	[0.93, 1.12]
South East	374	410.66	0.91	[0.82, 1.01]
South West	216	241.35	0.89	[0.78, 1.02]

STR = standardised test ratio; CI = confidence interval.

Observed is the number of positive cases in region; Expected is the number based on indirect standardisation. STR values >1 indicate higher than expected, <1 lower than expected.
