# Supplementary material for: Patterns of X-Linked Retinitis Pigmentosa Genetic Testing in England and Implications for Service Provision
Source: Ophthalmol Sci. 2026 Apr 1;6(6):101180. doi: 10.1016/j.xops.2026.101180 (PMC13127330; doi:10.1016/j.xops.2026.101180)
Supplement: Supplemental Table S3 [file mmc7.pdf]

Supplemental Table S3. Age and Sex Standardised Genetic Testing for RPGR-XLRP by IMD Quintile (2004-2024)

IMD Quintiles	Observed	Expected	STR	CI
1	603	596.17	1.01	[0.93, 1.1]
2	512	560.59	0.91	[0.84, 1]
3	498	507.72	0.98	[0.9, 1.07]
4	526	471.92	1.11	[1.02, 1.21]
5	455	457.59	0.99	[0.91, 1.09]

IMD = Index of Multiple Deprivation; STR = standardised test ratio; CI = confidence interval. Quintiles are based on national deprivation ranking (1 = most deprived, 5 = least deprived). STR values >1 indicate higher than expected, <1 lower than expected.
