# Supplementary material for: Patterns of X-Linked Retinitis Pigmentosa Genetic Testing in England and Implications for Service Provision
Source: Ophthalmol Sci. 2026 Apr 1;6(6):101180. doi: 10.1016/j.xops.2026.101180 (PMC13127330; doi:10.1016/j.xops.2026.101180)
Supplement: Supplemental Table S4 [file mmc8.pdf]

Supplemental Table S4. Prevalence Calculation

Section	Metric	Value
Input Parameters	Total diagnosed	2,594
	Patients with positive test	1,024
	Study Period	2004-01-07 to 2024-09-17
	2024 Population (overall)	58,620,764
Survival Estimates	Estimated surviving patients (total cohort)*	2,458
	Overall Estimated Survival rate (%)	94.79%
	Positive Estimated Survival rate (%)	95.45%
Overall Prevalence	Estimated surviving patients who tested positive	977*
	2024 Population	58,620,764
	Mortality-adjusted prevalence rate (per 100,000)	1.67
Male Prevalence	Estimated surviving male patients who tested positive*	626*
	2024 Male population	28,724,756
	Male Mortality-adjusted rate (per 100,000)	2.18
Female Prevalence	Estimated surviving female patients who tested positive*	351*
	2024 Female population	29,896,008
	Female Mortality-adjusted rate (per 100,000)	1.17
Unadjusted Prevalence	Unadjusted prevalence rate (per 100,000)	1.75 (n=1024)
	Male Prevalence (per 100,000)	2.18 (n=657)
	Female Prevalence (per 100,000)	1.28 (n=367)

* Rounded down
